# Back-Health-Related Physical Activity and Exercise Knowledge in Adolescents: A Cross-Sectional Study

**DOI:** 10.3390/children9091291

**Published:** 2022-08-26

**Authors:** Vicente Miñana-Signes, Manuel Monfort-Pañego

**Affiliations:** Academic Unit of Physical Education, Body Languages Didactics Department, Teacher Training Faculty, University of Valencia, Av. dels Tarongers, 4, 46022 Valencia, Spain

**Keywords:** physical education, knowledge, exercise, back health, adolescents

## Abstract

Knowledge is a determining factor for the development of postural habits; it could be considered as the first step in the establishment of changes. The aim of this study was to analyze the level of specific back-health-related physical activity and exercise knowledge in adolescents. A cross-sectional study was conducted on a sample of 1500 high school students between the ages of 13 and 18 (mean age = 15.18 ± 1.44). Students from the Valencian Community (Spain) were recruited with a confidence level of 95% and an accepted standard error of ± 2.53%. Self-report questionnaires were used to record back-health-related physical activity knowledge. The level of specific knowledge of back-health education related to physical activity and exercise in adolescents was low (X = 2.05 ± 2.26). Only 10.9% of the students passed the specific knowledge test, achieving a score equal or superior to 5. The boys’ average score was higher (X = 2.17 ± 2.31) than the girls’ (X = 1.94 ± 2.21) with statistically significant differences (*p* = 0.048). The level of specific knowledge increased with age (*p* < 0.001). Secondary school students show a low level of specific knowledge concerning back health. It is recommended that back care education be a part of school curriculum.

## 1. Introduction

Non-specific low back pain (LBP) is a serious, common health problem affecting a large section of all age groups of the world’s population [1], including children and adolescents [2]. A recent study carried out on adolescents found an overall prevalence of low back pain of 46.7% (95% CI: 44.27 to 49.11), reporting a prevalence of 42.0% (95% CI: 36.63 to 43.41) for boys and 58.0% (95% CI: 49.73 to 56.51) for girls, with a statistically significant difference [3]. For instance, in the Valencian Community, Spain, the level of prevalence of low back pain is 44.5% according to Miñana-Signes and Monfort-Pañego [4].

As far as low back pain is concerned, one of the most important tools to prevent LBP, or to minimize the frequency and severity of the symptoms once produced, is the acquisition of knowledge concerning back health [5].

Several studies have recognized the importance of improving the knowledge students have concerning fitness and health [6,7]. It is stated that better domain knowledge related to fitness and health, such as the valuation of its physical form, training objectives, and the application of FITT (frequency, intensity, time and type of exercise) may improve physical activity, hence improving active lifestyles [8,9] and therefore people’s health and quality of life.

For Limon et al. [10] there is an urgent need for health promotion programs which seek to increase knowledge in the field of back health in the education system. These must involve teachers, parents and the students themselves, in order to produce the necessary changes. Related to this, numerous studies involving the assessment of knowledge concerning back care in the school population concluded that back-care education programs in schools are an effective strategy for the conceptual development of the content of back health within the educational curriculum [11,12,13,14,15,16,17,18,19,20,21,22,23,24,25,26,27,28,29,30,31,32,33,34,35].

It is known that knowledge, per se, is probably not enough to change the habits and healthy behavior of individuals [36,37,38]; however, in order for habits to become the key element for the improvement of health care, and specifically back health, access to knowledge and information should be the first step in the teaching-learning process, to establish healthy physical-activity habits [39,40]. 

It is widely accepted that voluntary behaviors are influenced by the corresponding knowledge [41], and there are specific, validated and reliable instruments to assess postural habits at school age [42,43]. Related to this, education can contribute to the development of an improved lifestyle through improving physical fitness and the proper execution of daily activities [5].

Based on this, a body of knowledge concerning the proper use of the back is necessary to prevent back injuries [11,13]. According to a recent article [44], it is important to determine the level of specific knowledge concerning back health related to activity and exercise in students.

With regards to the evaluation instruments used to develop diagnostic studies concerning knowledge on back health in adolescents, we find few studies published with validity, reliability and with their respective psychometric analyses [5,45,46]. The use of a single set of postural metrics might provide clearer evidence about which treatments work best and why [47].

According to a theoretical framework, this study hypothesized that, in general, the level of knowledge of the adolescent population is low, and that this level of knowledge increases with age due to experience.

Therefore, this study aimed to analyze the level of specific back-health-related physical activity and exercise knowledge in adolescents, and to find out if age and gender could predict improvements in students’ knowledge.

## 2. Materials and Methods

### 2.1. Study Design

A cross-sectional study design was performed.

### 2.2. Ethical Statement

The management of the centers, each group tutor and the parents were informed about the study and gave their written consent. Moreover, we obtained institutional ethical approval from the Ethics Committee in experimental research at the University of Valencia (H1509086047576).

### 2.3. Subject Population

The study population consists of secondary school students from the Valencian Community, Spain (N = 247,714). The population was divided into strata, or segments, according to the provinces of the Valencian Community (Alicante, Castellón and Valencia). The sample under study was selected based on convenience of a non-probabilistic sampling during the academic years 2015–2016 and 2017–2018.

A representative sample of 1500 students from the Valencian Community (Spain) (Table 1) were recruited with a confidence level of 95% and an accepted standard error of ± 2.53%. Individuals were aged between 13 and 18 years old (mean age = 15.18 ± 1.44). 51.6% were boys (n = 771; mean age = 15.25 ± 1.43) and 48.4% were girls (n = 723; mean age = 15.10 ± 1.45).

Continuing with the descriptive data of the sample, the characteristics of the sample with respect to height, weight and BMI are detailed below.

Table 2 describes the characteristics of the sample with respect to height, weight and BMI.

#### Selection Criteria

The selection of these participants was made taking into account the following criteria:

1. Educational centers belonging to the Valencian Community (public and private schools).
2. Students belonging to both sexes.
3. Students aged between 12 and 18 years old.
4. Students with or without low back pain.

The grounds for exclusion were based on the following criteria:

1. Being absent on the day of the administration of the questionnaire.
2. Not completing the questionnaire correctly and not answering questions from the socio-demographic section.
3. Students with disabilities: lesions of the spinal cord (i.e., spina bifida, quadriplegia, etc.), Cerebral palsy, Down syndrome, autism, tumors, etc.

### 2.4. Instrument

In order to measure specific knowledge related to carrying out physical activity and exercise, individuals were required to complete a questionnaire to assess Health and Back Care Knowledge related to Physical Activity and Exercise (HEBACAKNOW-PAE) [45]. This questionnaire is made up of 13 multiple-choice questions with three possible options, only one of them being correct. The score scale is between −5 and 10 points (Equation (1)). The items refer to knowledge about physical conditioning, muscle strengthening and stretching and joint mobility. The score −5 was obtained when all the questions were answered incorrectly and the score of 10 when all the questions were answered correctly.

(1) $$P=10\cdot \frac{1}{N}\cdot \left(1\cdot A+0\cdot B-\frac{1}{2}\cdot F\right)$$

This questionnaire had been validated in a previous study among 230 Spanish students aged 13 to 18 years old, achieving good test-retest reliability; Cronbach’s Alpha being 0.80, and the intraclass correlation coefficient being 0.80 (*p* < 0.01) [45].

### 2.5. Data Collection

The questionnaires were completed during Physical Education (PE) classes using the Google Drive application in the computer rooms of the centers participating in the study. An experienced researcher presented the questionnaire to the students, explained the procedure and rules for filling in the survey, and personally dealt with all the queries that individuals had.

### 2.6. Data Analysis

Statistical analysis was conducted using IBM^®^ SPSS^®^ USA v.26. The level of significance was *p* < 0.05. The Kolmogorov Smirnov test assessed the normality of distributions. Descriptive statistics including means and standard deviations were performed to represent specific knowledge between genders and age groups. To assess the level of knowledge, we used a 10-point scale and percentiles for “global score”. Student’s t-test was applied to determine if the means of two sets of data (gender variable) were significantly different from each other. Analysis of variance (one-way ANOVA) was used to determine whether there were any statistically significant differences between the means of groups of ages. A backward stepwise (wald) binary logistic regression analysis was carried out to calculate the odds ratio (OR) and its 95% confidence interval (CI) between knowledge (dependent variable) and gender and age (independent variables). 

## 3. Results

### 3.1. Normality of Distributions

The Kolmogorov Smirnov test showed that the data followed a normal distribution with *p* > 0.05.

### 3.2. Specific Level of Knowledge

The sample of adolescents showed a low level of specific knowledge regarding health and back care education related to physical activity and exercise (X = 2.05 ± 2.26).

In general, only 10.9% (n = 164) of the students passed the specific knowledge test, achieving a score equal or superior to 5 points, 9.5% (n = 69) for the girls and 12.3 (n = 95) for the boys, while 89.1% of the students failed the test. In Table 3 you can see the students who passed the test based on age groups.

Based on the categorization of scores followed by the Spanish educational system, Table 4 shows the final grades. A total of 70 students completed the HEBACAKNOW- PAE, achieving a grade equal or superior to the classification “good”.

Regarding the contents related to the specific muscles involved in strengthening the back and back health (item 4), only 40% (n = 584) answered the item correctly.

Almost half of the students (46.1%; n = 673) correctly answered item 5 concerning the proper implementation of exercise to strengthen the lower back muscles.

Only 40% (n = 582) of the students correctly answered item 6 concerning the correct performance of isometric exercises to strengthen the abdominal muscles.

A third of the students (33.3%; n = 485), correctly answered item 7 related to the proper execution of exercises to strengthen abdominal muscles, and in particular concerning the position of the legs to protect the health of the back.

Approximately 40% (n = 577) of the students correctly answered item 8 related to the correct execution of exercises to strengthen abdominal muscles, and in particular on the degree of trunk flexion for the healthy development of trunk muscles.

Regarding the cool-down exercise contents (item 9), it was found that nearly 17% (n = 243) of the students knew the specific muscle to stretch to improve the health and care of the back.

Related to the concept of joint movements which are not recommended (item 10), such as hyperextension and hyperflexion of the spine, only 21.9% (n = 318) of the students were able to correctly answer the question.

An item-by-item summary is shown below (Table 5).

With regards to gender, the boys’ (X = 2.17 ± 2.31) average score was higher than the girls’ (X = 1.94 ± 2.212), with statistically significant differences (*p* = 0.048).

**Figure 1:**
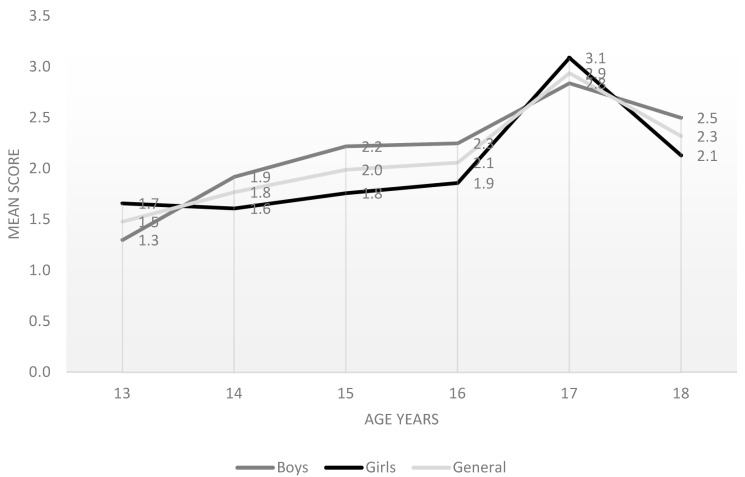
Mean scores by age groups and sexes.

The group of 17-year-olds obtained the highest scores. Except for the group of 18-year-olds, adolescents increased their knowledge on a regular basis, based on their increasing age, with statistically significant differences (F = 11.531; *p* < 0.001) (Figure 1).

### 3.3. Stepwise Binary Logistic Regression

This analysis showed that older students were significantly associated with a greater level of knowledge (Table 6). With regards to gender, no significant associations were found.

## 4. Discussion

This survey aimed to analyze the level of specific knowledge concerning health and back care related to physical activity in adolescents. A cross- sectional study was carried out, therefore it is not possible to demonstrate causality, but it is possible to show an association with variables. 

Students from the Valencian Community demonstrated that they have a very low level of specific back health related physical activity knowledge, scoring a mean of 2 points in the questionnaire. As seems obvious, the level of knowledge increased with age, perhaps due to the accumulation of experience, general knowledge and abilities [44,48].

The levels were so low that only 10.9% of the students passed the specific knowledge test, achieving a score equal or superior to 5, similar to another national and international studies [18,49,50]. However, a total of 70 students completed the test, achieving a score equal or superior to “good”. Among them, 30 students achieved a classification of “very good”, and 2 people managed to acquire the highest score. Therefore, we can say that it is possible to successfully attain a score of 50% and be familiar with the content related to back health and physical exercise.

With regard to gender, the boys scored significantly better than the girls. This could be because boys tend to play sports more often than girls [51,52,53,54], and they may be more informed about issues related to the practice of physical activity and exercise. In addition, physically active children were associated with physically active parents and friends, compared to inactive children [55], however, gender was not a predictor of good knowledge. Specific knowledge significantly increased with age. This seems obvious, since over time adolescents accumulate new experiences and learning.

With regard to the items, we can say that students have sufficient knowledge related to contents concerning physical qualities, frequency of exercise per week, the proper execution of lumbar strengthening exercises and the proper development and duration of stretching exercises. Gaps were observed related to the specific content and muscles involved in strengthening the back and back health, the proper execution of abdominal strengthening exercises (questioned in 3 items) and the specific muscles involved in the concept of joint movements which should be discouraged, such as hyperextension and hyperflexion of the spine.

In agreement with our results, most of the assessments carried out on the students concerning levels of knowledge about back health were found to be very low before the implementation of the intervention program [16,17,18,20,24,26,28,33,50,56]. Related to this, the results of studies that examined the level of knowledge in the field of physical education and health also drew attention to the low level attained by students, nowhere near the minimum level of proficiency in knowledge and understanding [49,57]. The results reinforce what Tellez stated [58] when he called attention to the fact that “students generally completely lack basic knowledge about the theory of the subject matter”. For these reasons, it is important to consider whether the content related to postural education is well-framed in the official curriculum, if teachers present said content in their classroom programs, and whether they have sufficient and appropriate knowledge about the subject [19,29,59]. On the other hand, and based on experience in the professional field of physical education teaching, we know that the PE is considered a highly practical subject. However, we think that the students could learn concepts through practice.

In assessment criteria, PE programming evaluates three aspects: concepts, procedures and attitude, with the conceptual area usually representing 20% of the mark, or less than the procedural content. In order to take advantage of this low percentage, a methodology that allows procedural contents to strengthen the conceptual contents, and vice versa, is required. To improve healthy habits in students, it is important for PE teachers to encourage the practice of physical activity and sport, but we must not forget that the subject also has an interdisciplinary and comprehensive character.

### 4.1. Limitations

As aspects to improve, or limitations of the study, it should be noted that the sample was recruited for convenience rather than randomization. On the other hand, the same data was collected over a period of two years and not over a shorter period of time. In addition, when the questionnaires were completed, it would have been interesting to differentiate between the students who had received previous training on back health or not.

### 4.2. Implications for Practice

From a welfare-oriented perspective, we want to know if the students’ knowledge concerning back health-related physical activity is related to the existence or absence of pain. In addition, we want to know if this could be interpreted as a preventive factor or indicator of risk, or if the students’ knowledge influences the acquisition of habits of active lifestyles, postural habits or engaging in regular organized physical activity.

Furthermore, from an educational point of view the assessment of the students’ knowledge is organized by the education system and prescribed in the official curriculum. For this reason, it is important to check what the students know, and can do, after completing their compulsory education [38]. Therefore, determining the actual knowledge possessed by students concerning health-related postural education at different levels of education directly involves more participation by professionals involved in PE, and, as a result, the use of measuring instruments to determine the degree of knowledge the students possess.

Meanwhile, to be able to assess what interventions related to health education and back care are needed (what the students do not know about back care and health), and also to check the effectiveness of the implemented interventions, it would be desirable to administer a complementary knowledge questionnaire [60] in order to assess the knowledge that students have about this topic, related to health and taking care of one’s back.

## 5. Conclusions

Most secondary students in the Valencian Community have demonstrated a poor or very poor level of specific knowledge concerning back health related to physical activity. The best results concerning knowledge were obtained by the oldest participants (17–18 years). Gender was not a predictor of knowledge; however, male participants did obtain better results than females. Physical Education teachers could review the postural education contents in their teaching programs and curriculums to ensure a better understanding and knowledge of these contents. The curriculum includes contents on the health of the back in secondary education, and teachers must ensure that these are achieved. This study serves to offer a good diagnosis of its needs.

## Figures and Tables

**Table 1:** Distribution of students by provinces and secondary education institutes.

Province	Secondary School	n	%
Alicante	SE n° 1	191	12.7
	SE n° 5	77	5.1
	SE n° 8	231	15.4
Castellón	SE n° 2	150	10.0
	SE n° 3	170	11.3
	SE n° 4	62	4.1
	SE n° 6	192	12.8
Valencia	SE n° 7	82	5.5
	SE n° 9	57	3.8
	SE n° 10	152	10.1
	SE n° 11	136	9.1
	Total	1500	100

SE: secondary school; n: frequency; %: percentage.

**Table 2:** Means and standard deviations of age, weight, height and BMI by sex.

	Boys n = 556		Girls n = 485	
	X	SD	X	SD
Age (years)	15	1.490	15	1.473
Weight (Kg)	62.71	9.169	53.53	9.169
Height (m)	1.67	0.010	1.67	0.099
BMI (Kg/m^2^)	20.78	3.007	20.96	3.268

n: frequency; X: mean; SD: Standard deviation.

**Table 3:** Distribution of the passed test based on age groups.

Ages	n	%
13 years	14	6.5
14 years	23	6.7
15 years	35	11.7
16 years	30	9.0
17 years	50	21.6
18 years	12	15.0
Total	164	100.0

n: frequency; %: percentage.

**Table 4:** Distribution of the students’ scores.

Scores	n	%
Very poor [≤2.9]	901	60.1
Poor [3–4.9]	435	29.0
Average [5–5.9]	92	6.1
Good [6–6.9]	38	2.5
Very good [7–8.9]	30	2.0
Excellent [≥9]	2	0.1
Total	1500	100.0

n: frequency; %: percentage.

**Table 5:** Summary of the scores obtained for each HEBACAKNOW-PAE item.

	Correct “1 Point”	
HEBACAKNOW-PAE items	n	%
Item 1. To take care of my back, what physical qualities should I pay special attention to and work on specifically?	1196	81.7
Item 2. How often should I do specific physical exercise to care for my back?	915	62.1
Item 3. To prepare my body to do a specific physical activity, what kind of exercises should I include in my warm-up?	949	64.6
Item 4. For back care, which muscles should we specifically strengthen?	584	39.9
Item 5. For the care of your back, which exercise to strengthen the trunk muscles is not being performed properly?	673	46.1
Item 6. For the care of your back, which exercise to strengthen the trunk muscles is not being performed properly?	582	40.2
Item 7. When we perform exercises to strengthen the abdominal muscles starting from the position of lying on our back, what is the correct position for the legs?	485	33.3
Item 8. When doing exercises to strengthen the abdominal muscles (abdominals) starting from the position of lying on my back, we must…	577	39.7
Item 9. For the health and care of my back, which muscles should I stretch specifically and with special attention?	243	17.0
Item 10. When I perform full extension or flexion movements of the trunk, what effects do they have on the lumbar spine?	318	21.9
Item 11. When doing a stretching exercise it is important	935	64.2
Item 12. How long does a stretch have to last?	882	60.3
Item 13. When I have discomfort in the lower back, I should	638	43.8

n: frequency; %: percentage.

**Table 6:** Backward stepwise binary logistic regression.

Variables	OR (95% CI)	*p*
13 years old	1	>0.001
14 years old	1.028 (0.517–2.045)	0.937
15 years old	1.884 (0.987–3.597)	0.055
16 years old	1.421 (0.735–2.748)	0.296
17 years old	3.927 (2.100–7.342)	>0.001
18 years old	2.508 (1.106–5.688)	0.028

OR: odds ratio; CI: confidence interval; *p*: level of critical significance.

## Data Availability

The data presented in this study are available on request from the corresponding author.
